# Taxonomic and Functional Differences in Cervical Microbiome Associated with Cervical Cancer Development

**DOI:** 10.1038/s41598-020-66607-4

**Published:** 2020-06-16

**Authors:** Charles Nkufi Tango, Sang-Soo Seo, Minji Kwon, Dong-Ock Lee, Ha Kyun Chang, Mi Kyung Kim

**Affiliations:** 1 Division of Cancer Epidemiology and Prevention, National Cancer Center, 323, Ilsan-ro, Ilsandong-gu, Goyang-si, 10408 Republic of Korea; 2 Center for Uterine Cancer, National Cancer Center, 323, Ilsan-ro, Ilsandong-gu, Goyang-si, 10408 Republic of Korea

**Keywords:** Cervical cancer, Cervical cancer

## Abstract

The cervical microbiome is associated with cervical cancer risk, but how microbial diversity and functional profiles change in cervical cancer remains unclear. Herein, we investigated microbial-compositional and functional differences between a control group and a high-grade cervical intraepithelial neoplasia and cervical cancer (CIN2/3-CC) group. After retrospective collection of 92 cervical swab samples, we carried out 16S rRNA amplicon sequencing on 50 and 42 samples from the control and CIN2/3-CC groups, respectively. The EzBioCloud pipeline was applied to identify the genomic features associated with the groups using 16S rRNA data. A linear discriminant analysis effect size (LEfSe) was performed to assess the enrichment in the assigned taxonomic and functional profiles. We found a lower richness in the control group relative to the CIN2/3-CC group; however, the β-diversity tended to be similar between the groups. The LEfSe analysis showed that a phylum *Sacchaaribacteria*_TM7, 11 genera, and 21 species were more abundant in the CIN2/3-CC group and that one uncharacterized *Gardnerella* species was more abundant only in the control group. Further characterization of the functional pathways using EzBioCloud showed that the 4 KEGG orthologs (Phosphotransferase system [PTS] sucrose-specific IIA, IIB, IIC components and PTS cellubiose-specific IIC component) were involved in the KEGG pathway of starch and sucrose metabolism. The two pathways of folate biosynthesis and oxidative phosphorylation were more abundant in the CIN2/3-CC group. Further confirmation of these results in larger samples can help to elucidate the potential association between the cervical microbiome and cervical cancer.

## Introduction

Cervical intraepithelial neoplasia (CIN) is a precancerous lesion in cervical epithelial cells, and is histologically divided into different grades^1^. Infection with HPV is the most important causative factor for CIN or cervical cancer. Although HPV is a major risk factor for cervical precancerous lesions or cancer, the potential role of the cervicovaginal microbiome in cervical cancer via the elevation of pH also has been reported^2^. In fact, the role of the cervicovaginal microbiome in HPV infection has already been established, which fact suggests a possible role in cervical cancer through potentiation of HPV infection^3^. Interestingly, the action of the microbiota is a complex process, the scientific data on which remains sparse^4,5^. The impact of the microbiome and its functions on cervical pathophysiology differ among individuals^6^. There is increasing evidence that the cervical microbiome plays important roles in the carcinogenesis process of the uterine cervix; thus, it is being considered as a target for development of innovative therapeutic approaches^7,8^. The cervical microbiome’s importance lies in its provision of the metabolic pathways and enzymatic machinery that help to process essential vitamins, remove toxic compounds, defend against pathogens, strengthen the female genital-tract epithelium, and stimulate as well as regulate the immune system. Previous studies have demonstrated that changes in the cervical microbiome might increase the risk of cervical carcinogenic progression^9–11^.

Currently, the most important approaches to the study of human microbiome changes associated with specific cancers are 16S ribosomal RNA (16S rRNA) amplicon and whole-metagenome shotgun sequencing^12^. Shotgun sequencing entails the analysis of the entire genomic content of a microbial community and provides insight into the taxonomic and functional profiles; however, it remains expensive, as it requires more extensive data analysis^13,14^. 16S rRNA amplicon sequencing, routinely performed, is a cost-effective approach to determination of microbial taxonomic composition, but it does not allow for direct functional assessments^12^. Fortunately though, specific pipelines such as the EzBioCloud 16S-based Microbiome Taxonomic Profile recently have become available for prediction of functional profiles using 16S rRNA sequencing information^15–17^. Pipelines predict the gene families that are present in a microbial community along with their relatively abundant pathways and orthologs^18^. Longitudinal studies of the microbiome during the CIN processes using metagenomic sequencing have indicated that host genetic variants can interact with the microbial composition, and that these genetic variants might be more abundant in cancer-cell-related pathways and orthologs^19^. In addition, recent dysbiosis studies have reported that progression of CIN to cancer is often accompanied by increased cervical microbiome diversity^9,20^.

Despite the initial interesting findings reported to the present time, still little is known about the complex interaction between cervical dysbiosis and cancer pathophysiology. To obtain insight into the contributory roles of the microbiome during cervical carcinogenesis, we investigated and compared the differences among women diagnosed with a high grade of CIN (CIN2/3), a cervical cancer group (CIN2/3-CC), and a control group. Specifically, we evaluated the microbiome diversity and taxonomic composition along with the related functional pathways and orthologs associated with risk of CINs and cervical cancer.

## Results

### General characteristics of study subjects

We performed a microbiome analysis, using 16 s rRNA amplicon sequencing, on 50 healthy subjects (control) and 42 patients diagnosed with cervical intraepithelial neoplasia grade 2/3 and invasive cancer (CIN2/3-CC). The demographic and lifestyle characteristics of the study participants are shown in Table 1. No significant inter-group differences were observed.

**Table 1:** Demographic and lifestyle characteristics of study subjects.

Characteristics	Control (n = 50)	CIN2/3-CC (n = 42)	*p*-value
Age (years)a	45.1 (11.6)	45.7 (11.7)	0.877
Body-mass index (Kg/m^2^)a	21.9 (2.8)	22.1 (2.9)	0.97
HPV status (%)			
Positive	27 (54.0)	29 (69.1)	0.141
Negative	23 (46.0)	13 (30.9)	
Menopause status (%)_b_			
Pre-menopause	29 (63.0)b	25 (62.5)	0.959
Post-menopause	17 (37.0)	15 (37.5)	
Smoking status (%)^b^			
No	37 (80.4)	35 (87.5)	0.376
Yes	9 (19.6)	5 (12.5)	
Alcohol-drinking status (%)			
No	15 (35.6)	6 (15.0)	0.058
Yes	31 (67.4)	34 (85.0)	
Oral contraception use (%)^b^			
No	38 (82.6)	33 (82.5)	0.989
Yes	8 (17.4)	7 (17.5)	

^a^For age and body-mass index, the values are in means (standard deviations).

^b^For menopause, smoking, alcohol-drinking status and oral contraception use, values are in frequencies (%). The *p*-value was calculated by chi-squared test for categorical variables and by *t*- test for continuous variables.

### Richness and diversity in cervical microbiome

**Figure 1:**
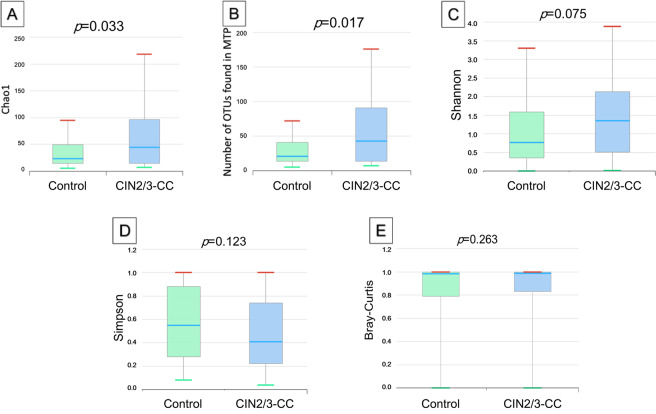
Comparison of species richness and α-, β-diversities in microbiome taxonomic profile between control and CIN2/3-CC groups by (**A)**. Chao1, (**B)**. Number of OTUs, (**C)**. Shannon, (**D)**. Simpson, and (**E)**. Bray-Curtis indices. The Shannon and Simpson α-diversity indices were applied to estimate the diversity for each group using the Wilcoxon rank-sum test. Beta diversity was calculated with Bray-Curtis distances based on the taxonomic abundance profiles. Permutational multivariate analysis of variance (PERMANOVA) was applied to measure the statistical significances of β-diversity.

We compared the microbiome richness and diversity between the control and CIN2/3-CC groups (Fig. 1). The microbial richness of cervical swabs evaluated at the species level was significantly higher in the CIN2/3-CC group than in the control group, as measured by Chao 1 (*p* = 0.03), and the number of operational taxonomic units (OTUs) found in the microbiome taxonomic profile (MTP) index (*p* = 0.017) was higher in the CIN2/3-CC group as well. Conversely, the diversity index, as calculated using the Shannon, Simpson, and Bray-Curtis indices, did not differ significantly between the groups at the phylum or genera level.

### Identification of cervical microbiome between control and CIN2/3-CC groups

We found that seven phyla (*Firmicutes*, *Actinobacteria*, *Bacteriodetes*, *Proteobacteria*, *Fusobacteria*, *Tenericutes*, and *Saccharibacteria*_TM7) were all highly abundant (with averages higher than 0.1%) in the cervical swab samples (Table 2). The phylum *Firmicutes* were most abundant in both groups. The phylum *Saccharibacteria*_TM7 was less abundant in the CIN2/3-CC group (*p* = 0.002) than in the control group. We explored the relative abundances of genus *Lactobacillus* in the groups using the Wilcoxon rank-sun test. However, none of the identified *Lactobacillus* species reached the level of significant difference between the control and CIN2/3-CC groups (Supplementary Fig. 1).

**Table 2:** Relative abundances of taxonomic composition of cervical microbiome at phylum level in study groups.

Taxon rank	Taxa name	Normal (%)	CIN2/3-CC (%)	*p*-value
Phylum	*Actinobacteria*	11.09	6.87	0.5870
	*Bacteriodetes*	7.54	10.79	0.0919
	*Firmicutes*	74.60	67.46	0.2098
	*Fusobacteria*	2.71	5.20	0.3515
	*Proteobacteria*	3.21	6.34	0.4710
	*Tenericutes*	0.79	2.72	0.4533
	*Saccharibacteria*_TM	0.04	0.50	0.0029
	7	0.02	0.61	
	ETC			

ETC: All taxa that are present in minor quantity (under 0.1% in average relative abundance). The p-value was calculated by Wilcoxon rank-sum test.

**Figure 2:**
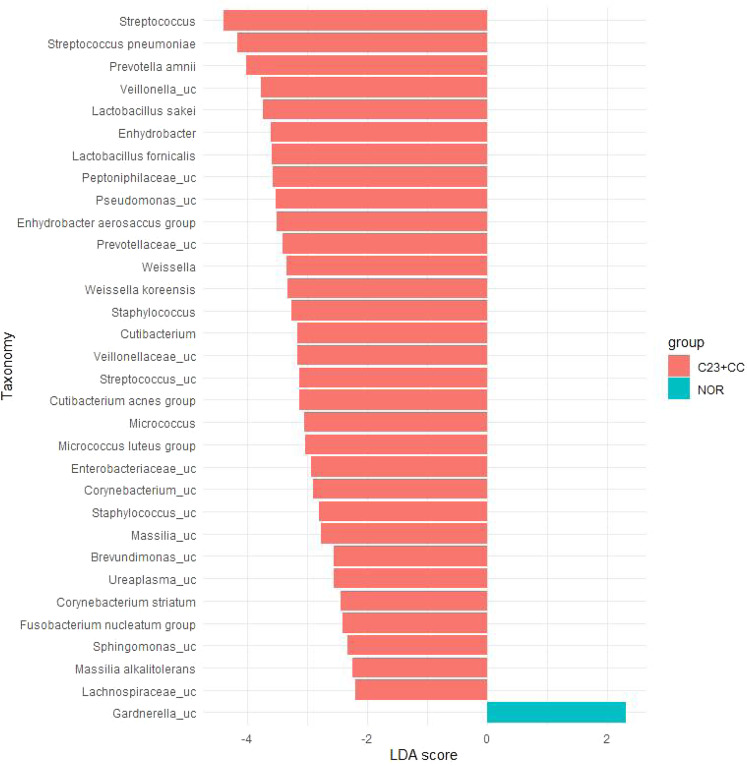
Differences in relative abundances of microbial taxa (genus and species) between groups by LEfSe analysis (Logarithmic LDA score >2.0; alpha value <0.05).

Differentially abundant taxa between the control and CIN2/3-CC groups were identified using linear discriminant analysis effect size (LEfSe) (minimum LDA score: 2.0) (Fig. 2). This analysis discovered 43 taxa, including one phylum and one class, 3 orders, 6 families, 11 genera, and 21 species, all of which were significantly abundant and discriminative between the groups (Table 3). The phylum *Saccharibacteria*_TM7, the *Saccharimonas* class, and the *Saccharimonas*, *Bacillales*, and *Propionibacteriales* orders were abundant in the CIN2/3-CC group. Moreover, the bacterial species *Streptococcus_*uc*, Massilia_*uc*, Ureaplasma_*uc*, Staphylococcus_*uc*, Fusobacterium nucleatum, Prevotella amnii*, and *Veillonella_*uc were highly abundant in the CIN2/3-CC group, with an FDR-adjusted *p*-value lower than 0.02. However, only one species, *Gardnerella*_uc, was significantly depleted in the CIN2/3-CC group relative to the control group (*p* value = 0.0013).

**Table 3:** Relative abundances of microbial taxa in control and CIN2/3-CCgroups. The statistical significance was tested using LEfSe analysis at the *p*-value of 0.05.

Taxon rank	Taxon name	*p*-value	Normal	CIN2/3-CC
Phylum	*Saccharibacteria_TM7*	0.00291	0.03892	0.49686
Class	*Saccharimonas_c*	0.00291	0.03892	0.49686
Order	*Saccharimonas_o*	0.00291	0.03892	0.49686
	*Bacillales*	0.00395	0.07537	0.45815
	*Propionibacteriales*	0.01669	0.08926	0.44539
Family	*Fusobacteriaceae*	0.00967	0.00593	0.21032
	*Streptococcaceae*	0.01148	1.78512	6.82772
	*Staphylococcaceae*	0.01419	0.03194	0.40041
	*Propionibacteriaceae*	0.01669	0.08926	0.44539
	*Saccharimonas_f*	0.02275	0.03892	0.47207
	*Lachnospiraceae*	0.04428	0.94424	3.50125
Genus	*Veillonellaceae_uc*	0.00301	0.00117	0.28635
	*Lachnospiraceae_uc*	0.00601	0	0.03159
	*Streptococcus*	0.01216	1.78512	6.79264
	*Micrococcus*	0.01263	0	0.22499
	*Enterobacteriaceae_uc*	0.01263	0	0.1733
	*Staphylococcus*	0.01419	0.03194	0.40041
	*Prevotellaceae_uc*	0.01841	0.09564	0.59931
	*Enhydrobacter*	0.02028	0.00368	0.79473
	*Weissella*	0.02653	0	0.43576
	*Peptoniphilaceae_uc*	0.02653	0	0.75755
	*Cutibacterium*	0.03125	0.08685	0.377
Species	*Gardnerella_uc*	0.00131	0.04132	0
	*Streptococcus_uc*	0.00159	0.0003	0.27272
	*Massilia_uc*	0.00601	0	0.11708
	*Ureaplasma_uc*	0.00758	0.00529	0.07693
	*Staphylococcus_uc*	0.01207	0.00027	0.12544
	*Fusobacterium nucleatum*	0.01263	0	0.05054
	*Prevotella amnii*	0.01297	1.09446	3.17349
	*Veillonella_uc*	0.01603	0.00083	1.15388
	*Enhydrobacter aerosaccus*	0.02028	0.00368	0.64059
	*Corynebacterium_uc*	0.02441	0.00041	0.16109
	*Lactobacillus sakei*	0.02653	0	1.08661
	*Weissella koreensis*	0.02653	0	0.4266
	*Corynebacterium striatum*	0.02653	0	0.05483
	*Brevundimonas_uc*	0.02653	0	0.07283
	*Micrococcus luteus*	0.02653	0	0.21356
	*Massilia alkalitolerans*	0.02653	0	0.03487
	*Sphingomonas_uc*	0.02784	0.00142	0.04329
	*Streptococcus pneumoniae*	0.02853	0.39632	3.28472
	*Cutibacterium acnes group*	0.02943	0.08332	0.35307
	*Pseudomonas_uc*	0.03192	0.01197	0.6767
	*Lactobacillus fornicalis*	0.04505	0.67222	1.4389

### Identification of metabolic-functional pathways between control and CIN2/3-CC groups

**Figure 3:**
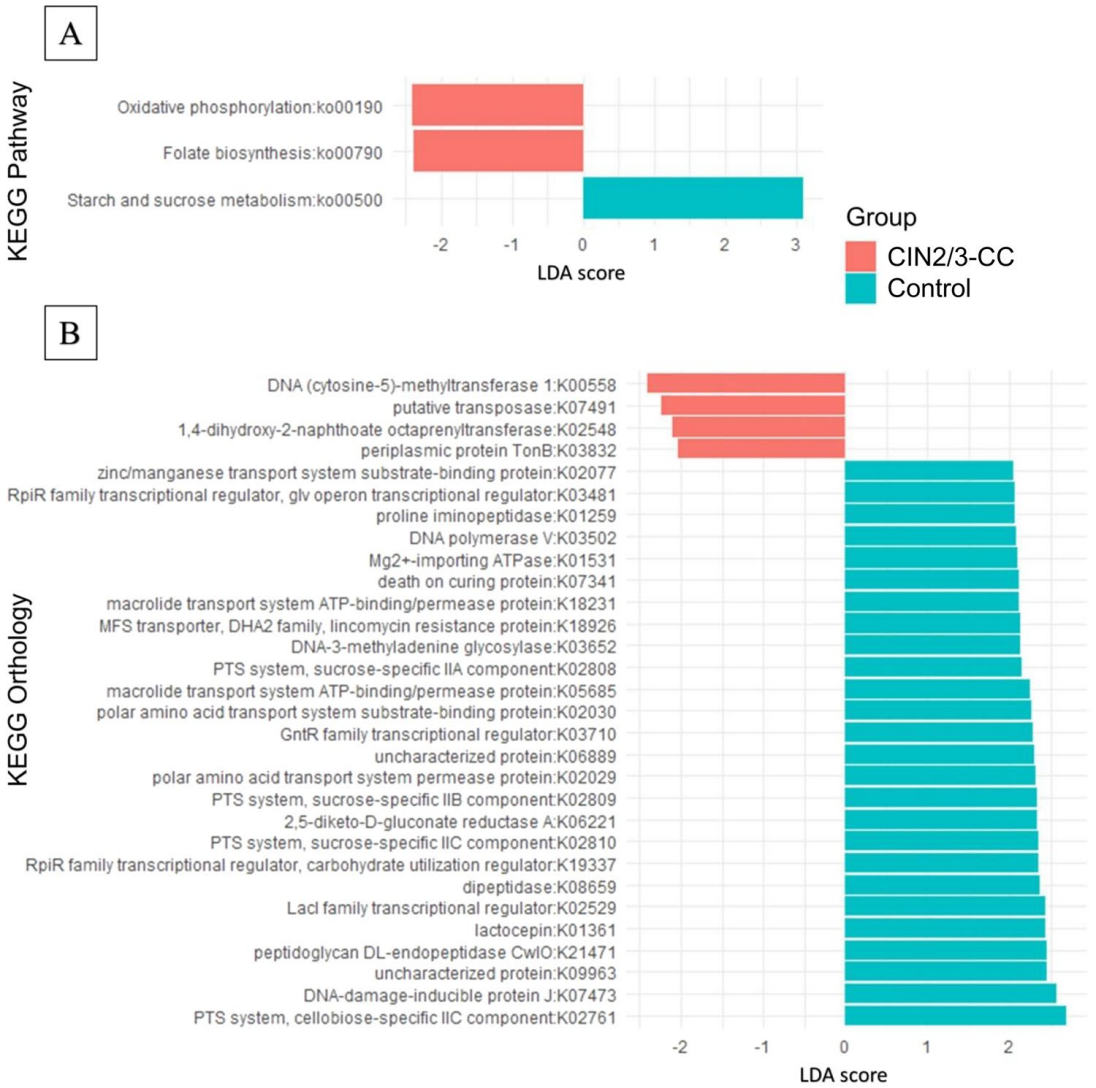
Differences in Kyoto Encyclopedia of Genes and Genomes (KEGG) pathway profiles between groups by LEfSe analysis (Logarithmic LDA score >2.0; alpha value <0.05). A. Pathway enrichment for KEGG cellular processes in control and CIN2/3-CC groups. B. Orthology enrichment for KEGG cellular processes in control and CIN2/3-CC groups.

We carried out a LEfSe analysis to discover the most relevant functional pathways responsible for the differences between the control and CIN2/3-CC groups. Among the 224 Kyoto Encyclopedia of Genes and Genomes (KEGG) pathways, three remarkably differed between the two groups (Fig. 3A). The starch and sucrose metabolism pathway (ko00500) was significantly (*p* = 0.02) abundant in the control group, while the KEGG pathways of oxidative phosphorylation (ko00190, *p* = 0.008) and folate metabolism (ko00790, *p* = 0.04) were significantly abundant in the CIN2/3-CC group. Of the 2860 KEGG orthologs (KOs), 30 showed significant inter-group differences (Fig. 3B). Among those 30 KOs, only 4 KEGG orthologs (i.e., DNA (cytosine-5)-methyltransferase 1, putative transposase, 1, 4-dihydroxy-2-naphthoate octaprenyl transferase, periplasmic protein TonB) were more abundant in the CIN2/3-CC group, whereas the remaining 26 were more abundant in the control group (Fig. 3B). Four of the KO (Phosphotransferase system [PTS] sucrose-specific IIA, IIB, IIC components and PTS cellubiose-specific IIC component) were involved in the KEGG pathway of starch and sucrose metabolism (ko00500) (Table 4).

**Table 4:** Mean relative abundances of 3 KEGG pathways and 4 KEGG orthologs (KO) involved in pathways identified using LEfSe analysis at the *p*-value of 0.05.

KEGG Pathway	Pathway Name	Control	CIN2/3-CC	*p*-value	Orthology	Definition	Control	CIN2/3-CC	*p*-value
ko00500	Starch and sucrose metabolism	1.5275	1.2775	0.0282	K02761	Phosphotransferase syst cellobiose-specificIIC	2.6781	0.2026	0.0250
					K02808	Phosphotransferase syst sucrose-specific IIA	2.1389	0.0573	0.0065
					K02809	Phosphotransferase syst sucrose-specific IIB	2.3309	0.1159	0.0456
					K02810	Phosphotransferase syst sucrose-specific IIC	2.3387	0.1158	0.0408
ko00190	Oxidative phosphorylation	0.8920	0.9423	0.0083					
Ko00790	Folate biosynthesis	0.4876	0.5349	0.0465					

## Discussion

To investigate the microbial dysbiosis associated with CIN progression to invasive cancer, we first evaluated the average relative abundances of phyla in the cervical microbiome. Secondly, we compared the diversity indices among the datasets to find whether the control group showed higher species diversity relative to the CIN2/3-CC group. Finally, we tried to identify differentially abundant taxa and metabolic pathways between the control and CIN2/3-CC groups. For compositional differentiation, we noted the appearance of the phylum *Saccharibacteria*_TM7 and the double augmentation of certain phyla such as *Bacteriodetes, Fusobacteria, Proteobacteria, and Tenericutes* in the CIN2/3-CC group compared with the control group. These results were supported by previous findings pointing to the overrepresentation of *Firmicutes* at low abundance in patients with cervical lesions and *Tenericutes*, which indicated an increase in proportion with increasing severity of CIN grade^19^. We observed significant depletion of certain phyla such as *Saccharibacteria*_TM7 in the control group relative to the CIN2/3-CC group. However, microbiome variation did not differ between the groups, either with regard to the diversity indices or sample variation (β-diversity). These results are supported by previous studies in which the bacterial diversities in the healthy group did not differ from those in CIN2/3-CC patients^9,21^. In the present study, we also found that species richness was higher in the CIN2/3-CC group than in the control group. In contrast to our results, an earlier study reported that bacterial richness was higher in women with high-grade CIN relative to healthy and low-grade CIN^22^. Microbial richness can be considered as an indicator of health status^12^, since variation of diversity can be associated with atypical health conditions. The higher richness observed in the present CIN2/3-CC group suggests that the bacterial diversity of cervical pre- and cancerous epithelial cells is partially altered from the healthy state^23^.

We also assessed whether selected *Lactobacilli* species could differentiate the CIN2:3-CC group from the control group. We observed that, although none of those bacteria reached statistical significance, *L. inners* showed a trend toward increased proportion, while the other identified *Lactobacillus* species, including *L. crispatus*, *L. fornicalis*, and *L. vaginalis*, tended to be less abundant proportions in the CIN2:3-CCgroup relative to the control group. Moreover, LEfSe analysis also demonstrated that an unclassified species of genus *Gardnerella* was more abundant in the control group. In line with our finding, another species of this genus (*Gardnerella vaginalis*) was associated with clearance of HPV infection^11^, which is the primary risk factor for cervical cancer. A study also reported that a cervical epithelium dominated by *L. iners* was associated with high-grade CIN^21^. In line with this, we found that 9 genera and 21 species were significantly abundant in the CIN2/3-CC group relative to the control group. Among them, the genera *Prevotella*, *Staphylococcus*, and *Streptococcus* were found to be positively associated with HPV infection persistence, precancerous lesion, and invasive cancer^19,24^. At the species level, *F. nucleatum* was shown to upregulate E-cadherin/a-catenin signaling through *FadA* adhesion and subsequently to promote colorectal cancer development^25^. *F. nucleatum* was found to modify the tumor-immune intestinal microenvironment and then to induce enteritis, colitis and inflammation associated with carcinogenesis^26^. Additionally, we discovered cervical bacterial taxa, including *Enhydrobacter aerosaccus*, *Corynebacterium striatum*, *Micrococcus luteus*, *Massilia alkalitolerans*, *Streptococcus pneumonia*, *Weissella koreensis*, and *Cutibacterium acnes*, which have not yet been reported.

Among the KEGG pathways, oxidative phosphorylation (ko00190) and folate metabolism (ko00790) were significantly abundant in the CIN2/3-CC group. The folate metabolism pathway has been reported to be essential in proliferating tissues, and one-carbon metabolism is upregulated in cancers^25^. Although the folate biosynthesis pathway has been found to be significantly altered in the fecal microbiome of prostate^27^ and cervical cancer patients, the biological function of folate in cervical carcinogenesis remains unclear. It might be linked with the activity and function of the fragile histidine triad and the 8-hydroxy-2’-deoxyguanosine gene^28^. However, there is also evidence that folate biosynthesis has an impact on different carcinogenesis processes, since certain anti-folate drugs, namely methotrexate and aminopterin, have been shown to be effective in treating cancers^29,30^. Therefore, enrichment of the folate biosynthesis pathway can be considered to be involved in cervical carcinogenesis. Further, it is commonly recognized that cell division exceeds the bioenergy demand through the process of aerobic glycolysis^31^. Due to the uncontrolled proliferation of cancer cells, the energy from aerobic glycolysis seems to be insufficient to support cellular metabolism in cell proliferation; therefore, cellular metabolism in cell proliferation is associated with, or even privileges, mitochondrial respiration through the oxidative phosphorylation pathway^32,33^. Pre- or cancerous cells use co-activator 1-alpha to boost the oxidative phosphorylation pathway^34^. Previous studies have reported that oxidative phosphorylation was more abundant in late-stage oral squamous cell carcinoma and breast cancer. Another study demonstrated that cells in colorectal cancer use the oxidative phosphorylation pathway to satisfy their metabolic demands^34^. In support of the above-mentioned findings, it is plausible that metabolic demand can explain the oxidative phosphorylation pathway enrichment of the microbiome in CIN2/3-CC group.

We also observed the enrichment of the starch and sucrose metabolism pathway in the control group compared with the CIN2/3-CC group. In line with our results, a recent study also demonstrated enrichment of the starch and sucrose metabolism pathway in healthy patients relative to those with prostate cancer^27^ and bladder cancer^35^. The starch and sucrose metabolism pathway was associated with phosphotransferase enzymes, including sucrose-specific IIA, IIB, and IIC, as predicted using the PICRUSt pipeline (Table 4). Instead of orthologs involved in the starch and sucrose metabolism pathway, we also identified certain orthologs that were more abundant in the control group, and that are involved in different biological processes. Interestingly, we found DNA polymerase *v*, which is often existent in many bacteria such as *Escherichia coli* as well as being involved in DNA replication, repair and damage tolerance. It has been reported that polymerase plays an essential role in trans-lesion synthesis in breast cancer cells^36^. We also found lactocepin, which is a lactic-acid-bacteria-secreted protein that has been shown to have a potential to degrade certain pro-inflammatory chemokines such as interferon-gamma-inducible protein 10^37^. It has also been demonstrated that lactocepin expressed by *Bifidobacterium* spp. can improve colitis associated with cancer in mice^38^. The PICRUSt pipeline has predicted, moreover, that DNA-3-methyladenine glycosylase, a DNA alkylation damage agent, has a cell-protective function against the killing effect of chloroethylnitrosoureas during cancer chemotherapy and also plays an important role in cancer prevention^39,40^. The control group in the present study also showed more abundant DNA-damage-inducible protein J, which belongs to a group of inducible proteins. These proteins have the main function of assuring the maintenance of the lesion repair process^41^. By contrast, we also predicted and profiled four orthologs that were more abundant in the CIN2/3-CC group relative to the control group, and two of them, DNA (cytosine-5)-methyltransferase 1 (DNCMT1) and putative transposase, were found to be involved in the DNA methylation and replication processes. DNCMT1 has been shown to have a potential for methylation-induced gene silencing as well as maintenance of CpG island methylation in human cancer cells^42,43^. As for the putative transposase, it has been demonstrated to act as an oncogenic mutator, and, therefore also, as a contributor to the development of a broad spectrum of tumors in human and mouse cancers^42,43^. The identification of these more abundant pathways and orthologs in the CIN2/3-CC group (not in the control group) was suggestive of a metabolism whereby changes are induced in cervical carcinogenesis.

In conclusion, in this case-control analysis of the cervical microbiome using 16 s rRNA amplicon sequencing with EzBioCloud, we observed significantly different microbial abundances and enriched metabolic functions between normal controls and CIN2/3-CC patients. The identification of certain species such as *F. nucleatum* and *P. amnii* and functional profiles (folate biosynthesis and oxidative phosphorylation) during CIN progression to cervical cancer might contribute to improved early diagnostics for patients with precancerous disease. Future studies should aim to elucidate the specific roles of bacteria, pathways and orthologs for enhanced understanding of the role of the cervical microbiome in cervical carcinogenesis.

## Materials and Methods

### Study design and subjects

The protocol for cohort study recruitment conformed to the Declaration of Helsinki and was approved by the Research Ethics Committee of the National Cancer Center (NCC) of Korea (IRB No. NCC2016-0147). From March 2006 to the present, the Korean HPV cohort study including women aged 18–65 years has been ongoing. Details on the cohort design criteria are available in a previous paper^44^. Informed consent was obtained from all of the participants. The enrolled patients had been diagnosed with CIN2/3 or cervical cancer during screening of the cervical tract in the project mentioned above. Potential enrollees with any history of immune-related diseases, diabetes mellitus, antibiotic therapy, or cancer therapy within three months were excluded.

### DNA extraction and pyrosequencing

Cervical samples were collected during medical examinations of individual patients by the NCC hospital. The swab samples were collected from 42 cases with CIN2/3(n = 25) or cervical cancer (n = 17) and healthy controls (n = 50). The swabs were immediately transported to the laboratory on ice and stored at -80 °C until further experimentation. Bacterial DNA extraction was performed using the Fast DNA Spin extraction kit (MP Biomedical, Santa Ana, CA, USA) according to the manufacturer’s instructions. DNA sequencing was performed using a Roche platform (Roche454 GS-FLX plus, Branford, CT, USA) to generate single-end reads at Macrogen Company Ltd. (Seoul, Republic of Korea). Pyrosequencing reads are available in the EMBL SRA database (http://www.ebi.ac.uk/ena/data/view/PRJEB5760).

### Quality-controlled 16S reads and taxonomic assignment

The single-end reads were uploaded to the EzBioCloud 16S-based MTP app (ChunLab, Inc., Seoul, Republic of Korea) to check the data quality. The cloud app of the Ezbiocloud software was used to detect and filter out sequences of low quality with regard to read length (<80 bp or >2,000 bp) and averaged Q values less than 25. Denoising and extraction of non-redundant reads were carried out using DUDE-Seq software. The UCHIME algorithm was applied against the Ezbiocloud 16S chimera-free database to check and remove chimera sequencing. Taxonomic assignment was performed using the USEARCH program to detect and calculate the sequence similarities of the query single-end reads against the EzBioCloud 16S database. EzBioCloud sequencing reads were clustered into OTUs at 97% sequence similarity using the UPARSE algorithm^45^. Single-end reads from each sample were clustered into many OTUs using the UCLUST tool with the above-noted cutoff values.

### Functional metagenome prediction

For the EzBioCloud 16S-based MTP pipeline, the PICRUST algorithm was used to estimate the functional profiles of the microbiome identified using 16S rRNA sequencing. The raw sequencing reads were computed using the EzBioCloud 16S microbiome pipeline with default parameters and discriminating reads that were encountered in the reference database. The functional abundance profiles of the cervical microbiome were annotated based on bioinformatics analyses, specifically by multiplying the vector of gene counts for each OTU by the abundance of that OTU in each sample^17^, using the KEGG (Kyoto Encyclopedia of Genes and Genomes) orthology and pathway database. The predicted metagenome profiles were categorized into clusters of KEGG Orthology and KEGG pathways and compared between the control and CIN2/3-CC groups. The accuracy of each of the functional profiles was determined according to the nearest sequenced taxon index^21^.

### Statistical and bioinformatic analyses

The differences in the demographic and lifestyle characteristics were examined between the groups using the *t*-test for continuous variables and the chi-squared test for categorical variables. Microbial richness was measured by Chao1 and the number of OTUs found in the microbiome taxonomic profile (MTP) index. The Shannon and Simpson α-diversity indices were applied to estimate the diversity for each group using the Wilcoxon rank-sum test. Beta diversity was calculated with Bray-Curtis distances based on the taxonomic abundance profiles. Permutational multivariate analysis of variance (PERMANOVA) was applied to measure the statistical significances of β-diversity. LEfSe was performed to determine enrichment in the assigned taxonomic and functional profiles of the two groups. Taxonomic levels with LEfSe values higher than 2 at a p-value < 0.05 were statistically significant. The ggplot2 package in the R program (version 3.4.3., R Foundation for Statistical Computing, Vienna, Austria) was used to visualize the LEfSe differences between the groups. All of the calculated *p* values were two-tailed and considered statistically significant at p < 0.05.

## Supplementary information

Supplementary table.

## Data Availability

The datasets generated during the current study are available in the EMBL SRA database (http://www.ebi.ac.uk/ena/data/view/PRJEB5760).
