# Supplementary material for: Taxonomic and Functional Differences in Cervical Microbiome Associated with Cervical Cancer Development
Source: Sci Rep. 2020 Jun 16;10:9720. doi: 10.1038/s41598-020-66607-4 (PMC7297964; doi:10.1038/s41598-020-66607-4)
Supplement: Supplementary file 1 — Supplementary table. [file 41598_2020_66607_MOESM1_ESM.docx]

Taxonomic and Functional Differences in Cervical Microbiome Associated with Cervical Cancer Development

Charles Nkufi Tango^1†^, Sang-Soo Seo^2†^, Minji Kwon^1^, Dong-Ock Lee^2^, Ha Kyun Chang^2^, Mi Kyung Kim ^1*^

^1^ Division of Cancer Epidemiology and Prevention, National Cancer Center, 323, Ilsan-ro, Ilsandong-gu, Goyang-si 10408, Republic of Korea

^2^ Center for Uterine Cancer, National Cancer Center, 323, Ilsan-ro, Ilsandong-gu, Goyang-si 10408, Republic of Korea

† These authors contributed equally to this work.

***Corresponding author**

Mi Kyung Kim, PhD

Division of Cancer Epidemiology and Prevention, National Cancer Center, 323, Ilsan-ro, Ilsandong-gu, Goyang-si 10408, Republic of Korea,

E-mail: [alrud@ncc.re.kr](mailto:alrud@ncc.re.kr), Tel: +82-31-920-2202, Fax: +82-31-920-2006


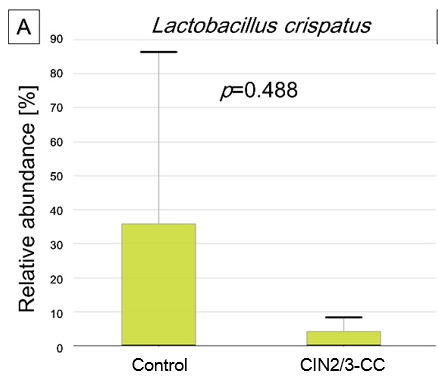

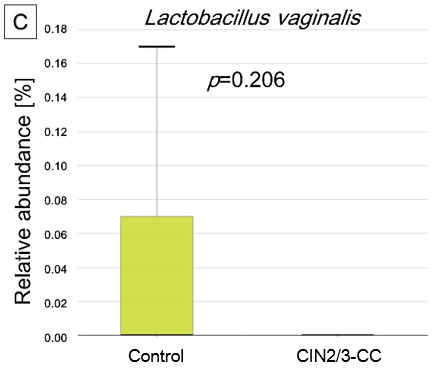

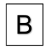

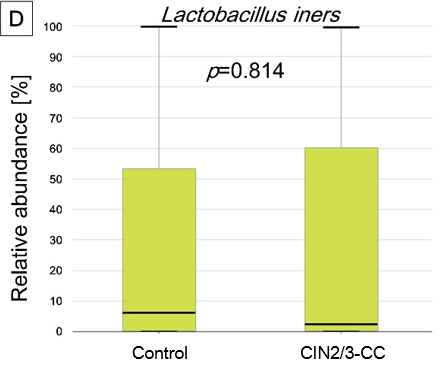

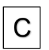


**Figure S1.** Relative abundances of selected *Lactobacillus* species. Comparison of identified *Lactobacillus* species between control and C2/3-CC groups. The Wilcoxon rank-sum test was used to measure the statistical significance at the *p*-value of 0.05.
